# Morphometry of the Entire Internal Carotid Artery on CT Angiography

**DOI:** 10.3390/medicina57080832

**Published:** 2021-08-17

**Authors:** Radu Andrei Baz, Cristian Scheau, Cosmin Niscoveanu, Petru Bordei

**Affiliations:** 1 Department of Radiology, Faculty of Medicine, “Ovidius” University, 900470 Constanta, Romania; raduandreibaz@yahoo.com; 2 Department of Physiology, “Carol Davila” University of Medicine and Pharmacy, 050474 Bucharest, Romania; 3 Department of Anatomy, Faculty of Medicine, “Ovidius” University, 900470 Constanta, Romania; bordei@anatomie.ro

**Keywords:** internal carotid artery, CT angiography, morphometry, anatomy, imaging study, anthropometry

## Abstract

*Background and Objectives*: Knowledge of the internal carotid artery’s (ICA) morphometric features is influential in outlining surgical and minimally invasive procedures in the neurovascular field. Many studies have shown divisive numbers regarding the ICA’s caliber, with the measuring point of the artery sometimes differing. This study presents ICA dimensions based on computed tomography angiography in each of its seven segments as per Bouthillier’s classification, correlating vascular dimensions with anthropometric parameters. *Materials and Methods*: A thorough CT angiography analysis was performed on 70 patients with internal carotid vessels unaffected by atherosclerotic disease. The extracranial part of the ICA was measured in four locations—carotid bulb, post-bulbar dilation, at its cervical midpoint, and below its entrance into the carotid foramen. Single landmarks were used for measurements in the intracranial segments. ICA length was assessed in the neck region and also in the cranial cavity. Craniometric measurements were performed on sagittal and coronal CT reconstructions. Patient height was taken into consideration. *Results*: The largest ICA portion is near its origin in the carotid sinus area (7.59 ± 1.00 mm), with a steep decline in caliber following its extracranial course. Distal ICA presented values somewhat similar to its proximal intracranial segment diameters (4.67 ± 0.47 mm). Dimensions of the ICA in the intracranial segments start from a value of 4.53 ± 0.47 mm and decrease by approximately 40% when reaching the origin of the middle cerebral artery (2.71 ± 0.37 mm), showing a marked decrease in caliber after the emergence of the most critical collateral artery, the ophthalmic branch. The length of the ICA varies between genders, with the male ICA being about 10 mm longer in total length than female ICA; this difference is also correlated with patient height and skull dimensions. *Conclusions*: Both intra- and extracranial ICA have variable dimensions and length related to gender and anthropometric parameters, with no significant differences obtained concerning side or age.

## 1. Introduction

Internal carotid arteries represent the main vessels responsible for the arterial vascularization of the brain, hence their importance in the context of circulatory disorders that may occur in this part of the body. Therefore, knowledge of their morphology, especially their origin and morphometric variants (caliber, length), is fundamental. The medical and surgical pathology of these vessels is quite complex, because they are a frequent site of atherosclerosis, stenosis, and aneurysms, especially in elderly patients [1].

Atherosclerosis in major arteries supplying blood to the brain is one of the most common causes of stroke worldwide. In 2019, 6.6 million deaths were attributable to stroke worldwide and several countries in Eastern Europe, Central and Southeast Asia, and Oceania reported the highest rates of stroke mortality. Each year, more females than males have a stroke, with females carrying a higher lifetime risk of stroke. The lifetime risk of stroke in populations 55 to 75 years of age is one in five for females and one in six for males [2].

Although rates of carotid endarterectomy (CEA) decreased between 1997 and 2014, the use of carotid artery stenting increased dramatically from 2004 to 2014. In-hospital mortality for CEA decreased steadily from 1993 to 2014 [2]. The difference in numbers is thought to be attributed to a better understanding of the maneuvers as well as the anatomic and physiologic factors included.

Given the frequency of vascular pathology on the carotid system, it is useful to master the anatomy of this system, as accurately and thoroughly as possible. The complete image of the supra-aortic trunks by computed tomography (CT) or magnetic resonance imaging (MRI) is necessary not only in the case of plaques developed in the bifurcation of the common carotid artery reported on ultrasonography or revealed by clinical symptoms, but also in a pre-interventional or pre-surgical context [3,4].

Direct observations, as well as detailed descriptions accompanied by numerical values, provide a high degree of accuracy and have led to a more accurate and useful approach to anatomical variants and abnormalities in medical practice [5,6]. It is well known that the atherosclerotic pathology of the carotid arteries exhibits clear links with anatomical factors [7], regardless of the individual risk factors; therefore, we aimed to detect diametrical variations in normal subjects, with no signs of atherosclerotic deposits on the supra-aortic trunks. There are numerous studies on the internal carotid artery in terms of its caliber [8,9,10,11], often with significantly different results due to different exploration methods but also due to the selected target site of measurement, even measuring arteries with marked atherosclerotic deposits. Koskinen et al. demonstrated a caliber of 4 mm/4.4 mm for female/male subjects using CT angiography; Krejza et al. determined diameters of 4.66 +/−0.78 mm and 5.11 +/−0.87 mm, respectively, using ultrasonography; and Wollschlaeger et al. measured ICA diameters of 3.88 mm on angiographic studies and 3.7 mm on cadavers [8,10,12]; all these measurements were performed above the carotid bulb. Additionally, the aforementioned studies have shown great variability regarding ICA caliber between sexes. CT angiography is a highly reproductive tool regarding measurements [13], and can provide fast, relevant, and thorough information regarding the ICA dimensions before surgical or minimally invasive interventions.

Precise knowledge of the carotid axis morphology may help clinicians in mapping out neck or facial surgery, endarterectomy, thrombectomy, tumor embolization, or other medical procedures, as well as thoroughly recording the presence and impact of severe stenosis, aneurysms, and other focal or generalized vascular anomalies [14,15,16,17].

We propose highlighting the morphometry of the internal carotid artery on CT angiographies for better knowledge of the anatomy of this vessel, including the caliber and length of the intracranial portion, according to Bouthillier’s segmentation [18], while also evaluating whether reported gender differences are, in fact, secondary to differences in the height and skull size of the patients.

## 2. Materials and Methods

We have performed a retrospective study between May 2020–May 2021 comprising 70 computed tomography angiography (CTA) images from the Radiology Department of “Sf. Apostol Andrei” County Hospital, totaling a number of 140 arteries. The subjects underwent CTA evaluation of the carotid arteries at the attending physician’s indication for various assessments in the context of their underlying disease. Prior informed consent has been signed.

Inclusion criterion for the study were the confirmation of normal ICA anatomy on CTA and, more precisely, the absence of atheroma plaques, and congenital or tumoral vascular pathologies involving the carotid vessels.

Examinations for cases of neck trauma, dissection, or vascular abnormalities were not included. Examinations in which a severe motion artifact was seen were excluded, in which case semi-automatic measurements were prohibited due to the lack of accuracy.

**Figure 1:**
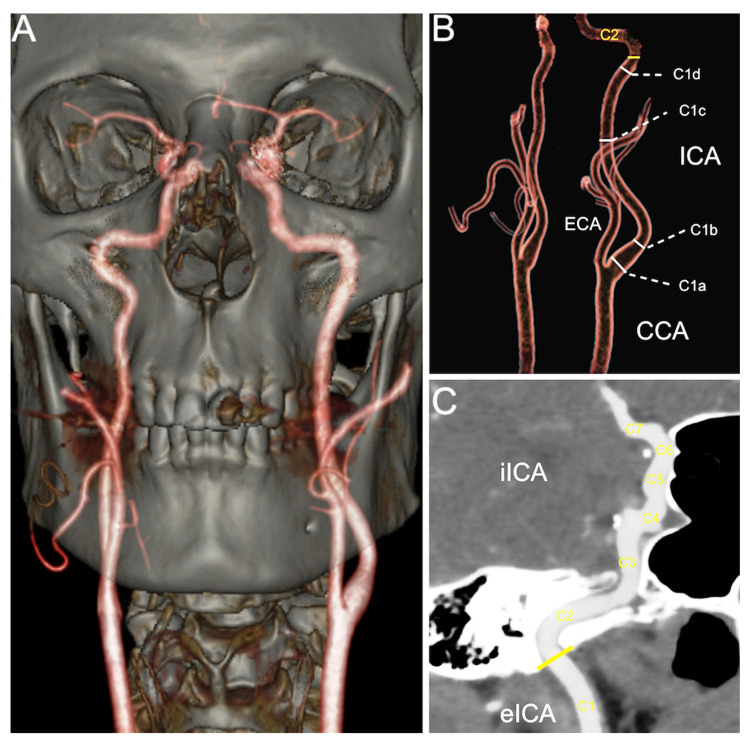
(**A**) VRT image of a carotid CT angiography; (**B**) VRT illustration of the cervical ICA measurement landmarks (carotid bulb—C1a; post-bulbar dilation—C1b; at the middle of C1—C1c; and below its entrance into carotid foramen—C1d) and representation of the carotid foramen position (yellow line); (**C**) oblique MIP image demonstrating set measurement points in the iICA (C2–C7). Abbreviations: CCA—common carotid artery; ICA—internal carotid artery; eICA—extracranial (cervical) part of ICA; iICA—intracranial part of ICA; ECA—external carotid artery; VRT—volume rendering technique; MIP—maximum intensity projection.

CTAs were performed on a multi-slice CT scanner with 64 detectors (LightSpeed VCT, General Electric Medical Systems, Chicago, IL, USA) using a section thickness of 0.625 mm, 0.984 pitch, 120 kV, auto-mA, and soft tissue reconstruction. The scan was enhanced by automatic intravenous injection of iodinated contrast material (Iomeron, 350 mg/mL) at a dose of 1–1.2 mL/kg body weight and an injection rate of 4 mL/s. The scan was initiated individually by tracking the loading bolus, with a region of interest located at the aortic arch level with automatic triggering at detected values ≥ 120 Hounsfield Units. From the source images, VRT reformations (volume rendering, see Figure 1) and MPR reconstructions (multiplanar reformatting) in the tri-planar system (axial, sagittal, and coronal) were performed. Image processing and postprocessing were conducted at a dedicated workstation (Advantage, General Electric Medical Systems, Chicago, IL, USA).

For analyzing the carotid arteries, we used semi-automatic vessel analysis software (Advanced Vessel Analysis, Version 4.2, General Electric Medical Systems, Chicago, IL, USA). The actual caliber was recorded by using a workstation add-on (VesselQ Xpress) with a built-in measurement tool of submillimeter accuracy (0.1 mm) from source images. For each ICA, the points of interest were set after careful examination of the entire ICA in three-dimensional views. Four target points were set in the extracranial portion of the ICA (carotid bulb—C1a, post-bulbar dilation—C1b, at the middle of C1—C1c, and below its entrance into carotid foramen—C1d) and one landmark for each of the intracranial segments, with the software determining the narrowest luminal diameter in millimeters (see Figure 1). If the lumen of the selected vessel mapped by the software and displayed as MPRs and reformatted curvilinear images had an unsatisfactory trace or noise, that examination was excluded from the study. The length of the ICA was measured using the same software. A total length measurement was achieved by setting a starting point at the bifurcation of the common carotid artery and an endpoint at the origin of the middle cerebral artery. The extracranial and intracranial lengths were measured according to the point of entry of the ICA in the carotid foramen, having the lower portion of the temporal bone as the reference point for the intracranial course.

Skull dimensions measured in our study were the height between the basion and the bregma (BBH), measured in the median–sagittal plane, and the bimastoid width (BiMa), measured in the coronal plane, as referenced in classic craniometric literature [19]. Patient height was retrieved from the digital records created on individual patient registration in the CT console.

All measurements were performed by two radiologists, one of whom was a resident doctor and the other a senior radiologist with experience in vascular imaging.

The statistical analysis was performed using MedCalc^®^ for Windows Version 20 (MedCalc Software Ltd., Ostend, Belgium). Student’s *t*-tests (two-tailed) were used to compare datasets between subgroups, with paired samples for left–right comparisons and assuming unequal variances for male–female comparisons. Pearson’s correlation coefficients were used to evaluate the relationship between craniometric measurements and patient height/age and the size of various vascular segments. Data were reported as means (±standard deviation). *p*-values were considered statistically significant at values below 0.05.

## 3. Results

The study group comprised 70 patients (33 male and 37 female) aged between 22 and 79 years old (56.66 ± 11.75, median of 59 years).

**Figure 2:**
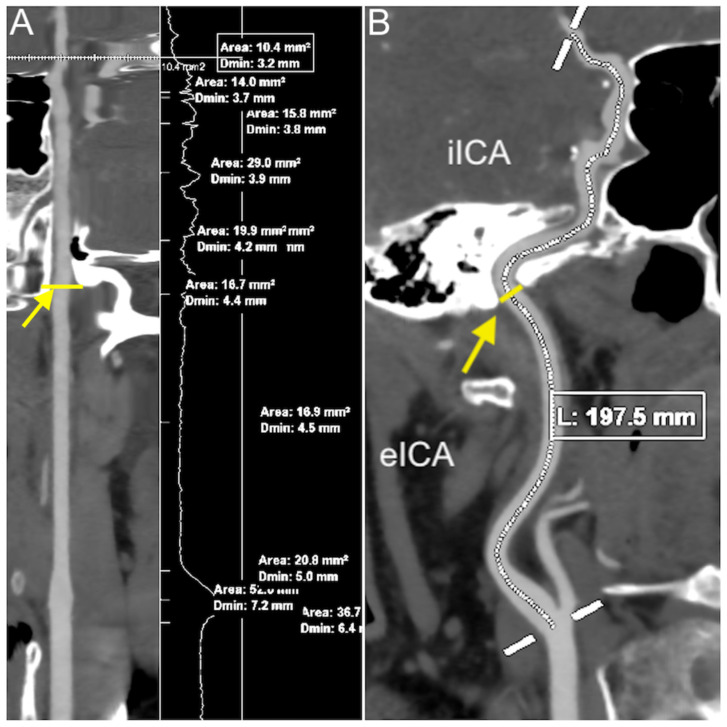
(**A**) Full measurement marks on a rendered stretched vessel image; (**B**) length measurement of the entire ICA on an oblique MIP image; yellow line and arrow in both images = entrance in the carotid foramen. Abbreviations: eICA—extracranial (cervical) part of the ICA; iICA— intracranial part of the ICA; MIP—maximum intensity projection.

The diameter of each segment of the internal carotid artery, as well as the length of the cervical and intracranial segments, were measured bilaterally for each patient (Figure 2). The detailed results are presented in Table 1.

The results show that the ICA had a decreasing caliber from the origin to its most distal part. In our study group, the average diameter of the intracranial segment of the ICA was 4.53 ± 0.47 mm in the C2 segment, which then decreased to 4.33 ± 0.45 mm in C3, then to 4.27 ± 0.45 mm in C4. A more significant diameter reduction was noticed after the emergence of the ophthalmic artery up to its terminal portion, where the ICA measures, on average, 2.71 ± 0.37 mm. Furthermore, regarding the C2–C7 segments, we did not find statistically significant differences related to laterality

Regarding the relationship between the patients’ age and the diameter of the internal carotid arteries or the length of the vessels, no significant correlations were identified.

In order to identify whether the differences between genders are, in fact, caused by differences in body size, we tested the correlation between vessel size (diameter and length) and patient height and skull size (Table 2).

**Figure 3:**
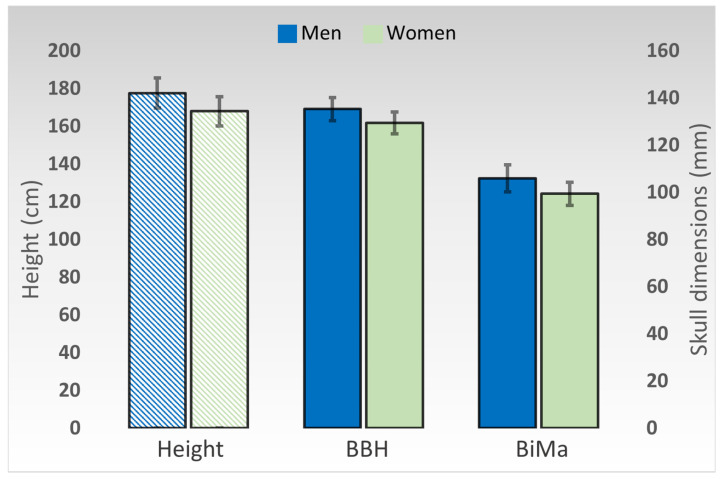
Graphical representation of the differences in average patient height and skull measurements between genders. Height is represented on the primary axis, in centimeters. Cranial diameters are represented on the secondary axis, in millimeters. Error bars represent standard deviations. BBH—height between the basion and the bregma. BiMa—bimastoid width.

Patient size and skull dimensions varied significantly between genders. The average height was 177.42 cm (±7.98) in men and 167.86 cm (±7.75) in women (*p* < 0.0001). BBH measured 135.15 mm (±4.82) in men and 129.30 mm (±4.69) in women (*p* < 0.0001), whereas BiMa measured 105.76 mm (±5.76) in men and 99.30 mm (± 4.93) in women (*p* < 0.0001) (Figure 3).

## 4. Discussion

The diameter of the internal carotid artery has been investigated since the 20th century in cadaveric studies and, later on, through angiographic studies [20,21]. In the last two decades, many more angiographic studies have been performed using newer imaging technologies such as ultrasonography, CT, and MRI [22,23]. The results of these studies are not always corroborated due to differences in the technology used, regarding the regions of interest or types of measurements.

With the introduction of semi-automatic caliber measurement software, the dimensions obtained became congruent regarding CT angiographies [13]. However, differences generated by the various measurement methods and the selected target still remain. For the extracranial internal carotid artery, studies in the literature report measurements either at the level of the carotid bulb where the dimensions are slightly larger, or immediately above the bulb, presenting lower caliber values. [24,25].

We presented four points of interest at the level of the extracranial ICA, measuring the artery at the level of the bulb, immediately after the bulb, in the middle of its cervical portion, and in its preforaminal portion, obtaining the results presented in Table 1.

No statistically significant differences were proven according to the studied part; the results were similar to those of other published studies using similar measurement techniques [12]. This finding may be useful in predicting minimal pathological changes to one side by using the other side as a normal reference.

A single key point of the caliber of each segment according to the Bouthilier classification was measured in the intracranial part of the internal carotid artery, going beyond the selective measurement locations accessible to ultrasound.

As mentioned in the Results section, ICA demonstrates a progressively reduced diameter that is more significant after the emergence of the ophthalmic artery. Our study group produced similar results to other morphometric studies [26,27,28,29]. Furthermore, regarding the C2–C7 segments, we did not find statistically significant differences related to laterality.

Bearing in mind that the prevalence of cerebral vascular events is higher in females, we compared the diameters of the ICA by gender, in all Bouthilier’s segments, and found significant differences between genders for almost all segments of the ICA (see Table 1). Another significant finding was that internal carotid arteries were shorter in the female population included in the study, both in the cervical segment of the vessel as well as in the intracranial portion (see Table 1). Although some studies have reported similar findings [25], others did not identify gender-related nor laterality-related differences in artery length [30].

The diameter and length of the internal carotid artery show significant gender-related variability, but also in relation to patient height and skull metrics. Therefore, the observed gender differences may in fact be caused by height and craniometric parameters. Similar findings were reported by Gabrielsen et al., who found a correlation between the dimensions of the ICA, gender, and skull width [21].

Using various models, some previously published studies have revealed that the vascular diameter may increase with age [31], including carotid trunks [32]. However, we did not find any significant increase in the arterial caliber of the ICA in older patients, although the aforementioned study did identify significant correlations with age [32].

In our approach, the presentation of morphometric aspects regarding the ICA on CT angiography in each of its seven segments may depict more accurate vascular measurements than cadaveric studies and cover more critical areas than ultrasonography, thus establishing a better understanding of carotid anatomy and paving the way for clear landmarks before and after neurological interventions. In some cases, precise knowledge of the reference ICA diameters may indicate the best type of endarterectomy to be employed while also offering a prognosis of changes in the blood flow, especially in women or some male patients with smaller vessel sizes [33,34]. Additionally, detailed morphometry of the entire ICA might facilitate more detailed devices and fitting accessories to be used in internal carotid procedures. Currently, a better stent fit may be obtained through tapering, but designing more size-adequate devices may facilitate deployment and orientation within the carotid vessels [35].

Our study had several limitations. We included patients without signs of vascular disease and with no major neurological events; thus, we cannot present any correlation with clinical signs or complications. Our study group focused on adult patients with a median age of 59 years old; therefore, from an age-related morphometric consideration, a broader group of patients is necessary to substantiate these findings. There are well-known difficulties in precisely measuring the terminal segments of the internal carotid artery due to bony structures in the vicinity and the small diameter after the emergence of the ophthalmic artery, also encountered by other studies using similar imaging methods [12]. Finally, the study group size was still relatively small compared to larger ultrasonographic studies, but this is also a limitation of the method.

## 5. Conclusions

The length, as well as the diameter, of most segments of the ICA vary significantly between male and female patients, but significant differences are also observed when skull dimensions and patient height are taken into consideration. Although age-related variations in the caliber of the ICA are reported in various studies, we did not identify any correlations between vessel diameter or length and patient age. Thorough knowledge of the normal morphometry of the internal carotid artery is essential for understanding clinical symptoms, the planning of vascular or perivascular interventions, as well as the development of applications, devices, or accessories used in various endovascular procedures.

## Figures and Tables

**Table 1:** CT angiography morphometric measurements of the entire ICA for all studied patients, stratified by laterality and gender. Results are expressed in millimeters (±standard deviation).

Segment	Right Side	Left Side	*p*-Value	Male	Female	*p*-Value
Carotid bulb (C1a)	7.56 (±0.97)	7.61 (±1.01)	0.6092	7.94 (±1.05)	7.27 (±0.82)	0.0001
Post-bulbar section (C1b)	5.49 (±0.64)	5.52 (±0.62)	0.6183	5.74 (±0.67)	5.31 (±0.50)	0.0001
Midpoint of C1 (C1c)	4.84 (±0.54)	4.85 (±0.53)	0.7750	5.01 (±0.56)	4.70 (±0.46)	0.0006
Endpoint of C1 (C1d)	4.65 (±0.46)	4.69 (±0.47)	0.4373	4.81 (±0.50)	4.55 (±0.40)	0.0013
C2	4.53 (±0.43)	4.53 (±0.50)	0.8731	4.66 (±0.50)	4.42 (±0.41)	0.0026
C3	4.32 (±0.41)	4.34 (±0.49)	0.7105	4.48 (±0.48)	4.20 (±0.38)	0.0002
C4	4.25 (±0.41)	4.28 (±0.48)	0.5618	4.43 (±0.46)	4.13 (±0.38)	0.0001
C5	4.13 (±0.46)	4.17 (±0.50)	0.3886	4.34 (±0.46)	3.98 (±0.43)	<0.0001
C6	2.89 (±0.38)	2.88 (±0.41)	0.4700	2.91 (±0.41)	2.86 (±0.38)	0.5307
C7	2.72 (±0.35)	2.70 (±0.38)	0.2770	2.73 (±0.37)	2.70 (±0.37)	0.6190
Extracranial length	86.33 (±18.76)	85.87 (±18.53)	0.5540	90.21 (±20.66)	82.43 (±15.76)	0.0151
Intracranial length	69.40 (±8.95)	69.33 (±8.42)	0.8370	71.29 (±7.97)	67.64 (±8.93)	0.0122
Total ICA length	154.89 (±26.17)	155.20 (±23.57)	0.7895	160.62 (±28.56)	150.07 (±19.83)	0.0141

**Table 2:** Correlations between vessel caliber and length vs. body and cranium size in the study cohort.

Segment	Measurement ^1^	Height	BBH ^2^	BiMa ^3^
Carotid bulb (C1a)	7.59 (±0.99)	*p* = 0.0005	*p* = 0.0080	*p* = 0.0226
Post-bulbar section (C1b)	5.51 (±0.63)	*p* = 0.0356	*p* = 0.4014	*p* = 0.5521
Midpoint of C1 (C1c)	4.85 (±0.53)	*p* = 0.0365	*p* = 0.0730	*p* = 0.1512
Endpoint of C1 (C1d)	4.67 (±0.47)	*p* = 0.0224	*p* = 0.1031	*p* = 0.1539
C2	4.53 (±0.47)	*p* = 0.0598	*p* = 0.3029	*p* = 0.2993
C3	4.33 (±0.45)	*p* = 0.0150	*p* = 0.1846	*p* = 0.1744
C4	4.27 (±0.45)	*p* = 0.0075	*p* = 0.0889	*p* = 0.0984
C5	4.15 (±0.48)	*p* = 0.0027	*p* = 0.0166	*p* = 0.0506
C6	2.88 (±0.40)	*p* = 0.4606	*p* = 0.1686	*p* = 0.2540
C7	2.71 (±0.37)	*p* = 0.3660	*p* = 0.3329	*p* = 0.6489
Extracranial length	86.10 (±18.65)	*p* < 0.0001	*p* = 0.0052	*p* = 0.0197
Intracranial length	69.36 (±8.69)	*p* < 0.0001	*p* < 0.0001	*p* < 0.0001
Total ICA length	155.04 (±24.90)	*p* < 0.0001	*p* < 0.0001	*p* = 0.0004

^1^ Measurements are presented as the average size in millimeters ± standard deviation; ^2^ height between the basion and the bregma; ^3^ bimastoid width.

## Data Availability

The data presented in this study are available on reasonable request from the corresponding author (C.N.).
